# A Bibliometric Analysis of Consumer Neuroscience towards Sustainable Consumption

**DOI:** 10.3390/bs13040298

**Published:** 2023-03-31

**Authors:** Yan Liu, Rui Zhao, Xin Xiong, Xinyun Ren

**Affiliations:** Faculty of Geosciences and Environmental Engineering, Southwest Jiaotong University, Chengdu 611756, China

**Keywords:** consumer neuroscience, bibliometric analysis, sustainable consumption, ERP, fMRI, machine learning

## Abstract

Consumer neuroscience is a new paradigm for studying consumer behavior, focusing on neuroscientific tools to explore the underlying neural processes and behavioral implications of consumption. Based on the bibliometric analysis tools, this paper provides a review of progress in research on consumer neuroscience during 2000–2021. In this paper, we identify research hotspots and frontiers in the field through a statistical analysis of bibliometric indicators, including the number of publications, countries, institutions, and keywords. Aiming at facilitating carbon neutrality via sustainable consumption, this paper discusses the prospects of applying neuroscience to sustainable consumption. The results show 364 publications in the field during 2000–2021, showing a rapid upward trend, indicating that consumer neuroscience research is gaining ground. The majority of these consumer neuroscience studies chose to use electroencephalogram tools, accounting for 63.8% of the total publications; the cutting-edge research mainly involved event-related potential (ERP) studies of various marketing stimuli interventions, functional magnetic resonance imaging (fMRI)-based studies of consumer decision-making and emotion-specific brain regions, and machine-learning-based studies of consumer decision-making optimization models.

## 1. Introduction

Carbon neutrality has become a commonly pursued value in the global response to climate change [1]. The achievement of this goal depends not only on cleaner energy sources and low-carbon technologies but also on the pro-environmental behavior of the public, that is, behaviors of sustainable consumption and production that synergistically promote reductions in emissions [2]. Research has shown that pro-environmental consumption may help increase the market share of green products and force companies to produce green innovations that improve the sustainability of their products and services [3].

Numerous factors influence pro-environmental behavior, including product or service attribute stimuli (such as logos, prices, packaging, etc.) [4]; individual consumer characteristics (such as sex, age, level of education) [5]; and social interventions (such as social paradigms, social expectations, and so on) [6,7]. The above factors are generally identified through research methods such as questionnaire surveys and in-depth interviews. The former is characterized by large sample sizes and, through path analysis and structural equation modeling, mainly reveal consumers’ purchase intentions and “willingness to pay” (WTP) as well as the relationships between these and related influencing factors [8,9]. The latter employ a relatively small sample size and are characterized by a large amount of information; they are mainly used to discuss the potential influencing factors affecting consumers’ purchase intentions and WTP [10]. However, these two types of research methods rely on respondents’ existing subjective perceptions and on the influences of the appraisal of others, which may lead to deviations between respondents’ expressed willingness and their real psychological activities [11]. In addition, as there is a certain element of time delay in the data samples collected through questionnaires and interviews, this may lead to unintended consequences of the surveys [12]. Emerging neuroscience techniques, through the combined application of neurophysiological and biological research methods, provide access to the physiological responses to consumer decision-making processes [13]. Based on electroencephalogram (EEG) signals, these techniques help to better understand implicit and underlying mechanisms [14]. For example, studies have shown that while 80% of consumers surveyed indicated a willingness to purchase green products, only 26% of consumers regularly purchased green products [15]. This reflects that, even though green consumption emphasizes the role of ethical norms in guiding awareness of climate change, there is often a large discrepancy between consumers’ attitudes and actual behaviors [16].

Studies have reported the progress of neuroscientific tools, such as EEG and functional magnetic resonance imaging (fMRI), in product pricing and brand preference [17]. This explains the psychological and brain area reflections generated by changes in consumers’ behavioral decisions. By reviewing existing studies, we are able to explore the research frontiers in the field of consumer neuroscience, identify research hotspots, and illuminate a path toward the promotion of applications of neuroscience in sustainable consumption. This study utilizes bibliometric analysis tool to review the progress of research in consumer neuroscience during the period 2000–2021. The statistical analysis of bibliometric indicators—including number of publications, countries, institutions, and keywords—was used to identify research hotspots in the field of consumer neuroscience. In response to carbon neutrality, this paper focuses on the prospects of future research on the application of neuroscientific tools to sustainable consumption.

The paper is structured as follows: Section 2 presents our data sources and techniques of data cleansing; Section 3 presents the results of the statistical analysis of bibliometric indicators—including number of publications, countries, subject categories, publishing journals, institutional distribution, highly cited papers, and keywords; Section 4 explores thoughts on the application of neuroscience to sustainable consumption; Section 5 discusses the research hotspots in consumer neuroscience and; and Section 6 provides overall conclusions and prospects for future research.

## 2. Materials and Methods

The study applies bibliometric analysis to uncover research trends in the field of consumer neuroscience by structuring the massive data into various categories, i.e., number of publications, institutional collaborations, keywords occurrences, etc., ultimately to map the research progress as well as identify emerging ideas for future study. Apart from other common review methods, including meta-analysis and systematic review, the bibliometric analysis is highlighted by its quantitative features that mainly focus on revealing the interaction among various bibliometric indicators [18]. However, this study embeds the characteristics of a systematic review into the bibliometric analysis, since the scope is confined to consumer neuroscience. When taking in-depth analysis of the bibliometric indicators, e.g., institutional collaboration, keywords, etc., the model of systematic review can reveal the development of consumer neuroscience through a summarization of similar studies.

Bibliographic data for this study were obtained from predefined searches in the Web of Science Core Collection database, from the Science Citation Index Expanded, Social Sciences Citation Index, and Arts and Humanities sub-databases. Searching for the terms “consumer” and “neuroscience” in the title, abstract, or index terms, a total of 364 papers were retrieved for the period 2000–2021. The TS search formula was as follows: [TS = (neuroscience) AND TS = (consumer) AND DT = (Article OR Review) AND DOP = (2000-01-01/2021-12-31)]. This specific search period was chosen because the concept of “consumer neuroscience” was first introduced by Smith in 2002 [19]. Based on the identified literature, we used the CiteSpace software to construct a bibliometric network to extract keywords from paper titles and abstracts, establish keyword co-occurrence relationships, and visualize emergent keywords to analyze trends of change in research [20].

## 3. Results

### 3.1. Number of Publications

**Figure 1:**
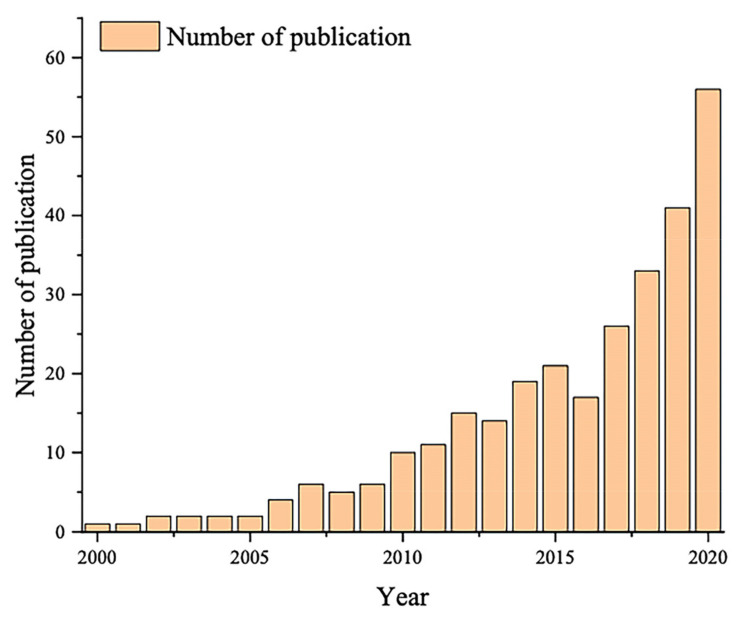
Publications output, 2000–2021.

As shown in Figure 1, within the field of consumer neuroscience research, a total of 364 papers were published between 2000 and 2021, showing significant growth. In particular, since 2019, there has been a sudden increase in the number of papers published on the topic, reflecting that the field is gradually becoming a focus of consumer research. Between 2000 and 2010, the number of papers published in this field remained stable at 10 papers annually or fewer, which can be considered as the initial stage of consumer neuroscience research. The focus of this phase was on establishing a research framework for consumer neuroscience while exploring neuroimaging tools (EEG, fMRI, etc.) and the application of these in evaluating areas of the brain that are active in consumers’ responses to a variety of marketing stimuli (including advertising, price, branding, etc.) [21,22]. Starting in 2010, the number of annual publications increased to more than 10, and in the following decade, the number of publications increased steadily annually, hovering at approximately 20–30 per year. This phase started with a large number of studies that used event-related potential (ERP) observations of the cognitive processes related to consumer attention, attitudes and preferences, decision-making and emotions, and so on [23]. There was a steep increase in the number of publications from 2018, reaching 70 articles a year by 2021. This shows that the research tools of neuroscience have reached a relative maturity while also providing effective predictive analysis of consumer patterns of behavior through the introduction of data analysis tools such as machine learning.

### 3.2. Country Cooperation Network

**Figure 2:**
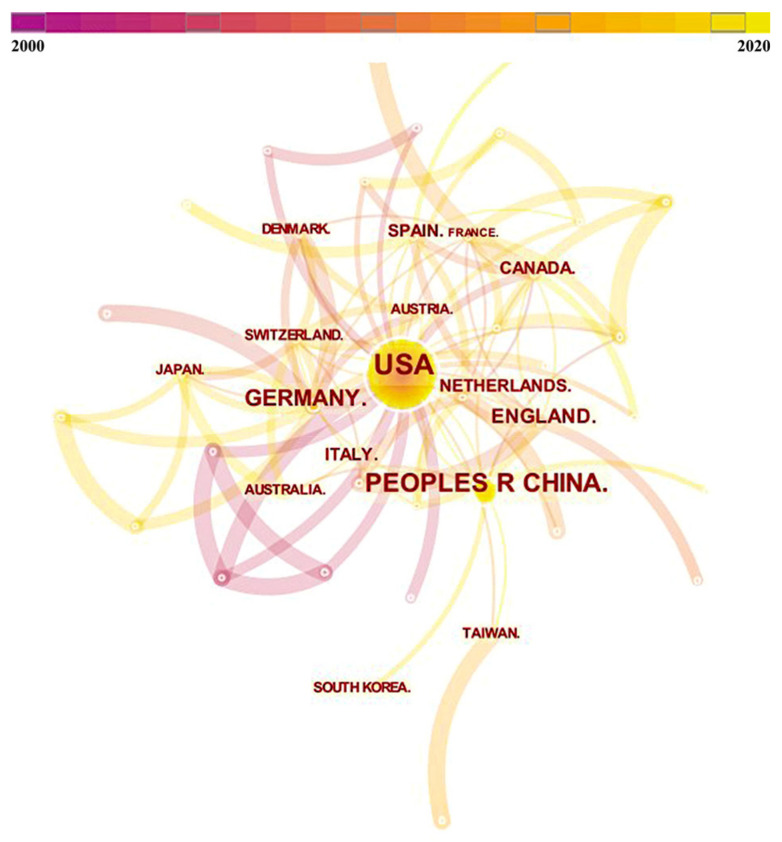
Inter-country cooperation network.

From 2000 to 2021, the network of collaborating countries consisted of 53 nodes and 104 links, as shown in Figure 2. The United States has the most papers published in the network, with 145 papers, followed by China (62 papers) and Germany (38 papers). The U.S. published papers mainly employed magnetic resonance imaging (MRI) techniques in combination with neuroimaging to highlight the impacts of the brand, mall environment, price, and public policy on consumer decisions in terms of different brain activation areas. During the same period, the U.S. had a relatively strong centrality within the network (0.46), indicating the strong radiative and correlational nature of its research output. Research papers published in China focused on changes in ERPs generated by consumer emotions stimulated by brands as well as online and offline payment methods. Germany explored consumer neural mechanisms triggered by the external environment (e.g., friends’ appraisal, social recognition, social media engagement, etc.) in addition to studying changes in consumer emotions, decision-making, and attention. Although China was higher than Germany in terms of the number of publications, the centrality of China (0.21) was lower than that of Germany (0.28), indicating that there is still room for China to explore international collaborations in the field of consumer neuroscience research. In addition, U.S. partnerships were mainly established after 2010, while Chinese partnerships were mainly established after 2015. This also correlates with the changing character of the number of publications, that is, the deepening of research in the field since 2010.

### 3.3. Institutional Cooperation Network

A total of 336 scientific institutions published research papers related to consumer neuroscience in the period 2000–2021. Given the large number of institutions (above 97%) with only 1–2 papers published, we included only those research institutions that are first authors and that have more than five publications, as shown in Table 1. Among them, a total of five universities and research institutions in the U.S. were selected (occupying 50%), followed by four universities in China. In addition, one university in the Netherlands was selected. Zhejiang University in China has the largest number of articles, with 14 articles focusing on the application of EEG techniques in analyzing consumer purchase intention, with ERPs P300 and late positive potential (LPP) as representative signals [24]. The contents of these studies were mostly focused on brand extension, online shopping advertisements, online payment methods, and selling prices. Among these, “brand extension” refers to the act of entering a new product category into an existing brand; if the product attributes are incompatible with the brand category, this may generate greater cognitive confusion, eliciting a relatively small P300 [25] and large N400 [26]. When shopping online, positive advertising messages tend to evoke larger LPP magnitudes in consumers, while negative messages may lead to larger P200-N200 complexes, evoking difficulties in decision-making [27]. Consumer adoption of mobile payments for online shopping generates larger N200 amplitudes and smaller N270 amplitudes [28]. Additionally, the sales price triggers N200 weakening and LPP increase [29]. The California Institute of Technology in the U.S. focused on neural activation related to decision-making and on the marketing placebo effect. The former, mainly based on MRI techniques, found that activity in the common areas of the orbitofrontal cortex (OFC) and ventral striatum showed strong neural activity in response to different marketing stimuli, and especially that positive feedback in consumers mainly activated the striatum and medial prefrontal cortex (MPFC), while negative responses mainly activated the insula and amygdala [30,31]. The latter focuses on the sustained effects of brand and price-based marketing stimuli on consumer experience and subsequent consumer behavior through brain imaging techniques. This is defined as the “marketing placebo effect” [32]. Research at Erasmus University in the Netherlands has focused on the neural responses generated by different advertisements and on consumer behavior under different social influences [33,34]. For example, female consumers are more likely to purchase products endorsed by celebrities, with medial orbitofrontal cortex (mOFC) activity increasing significantly, as observed using the fMRI [35]. Pozharliev et al. mentioned that significant brain activation in the occipital region is produced in consumers when they are influenced by the interactions with the people around them, such as when they watch advertisements for branded products together [36].

### 3.4. Keywords

Keywords can be used to reflect the research “hotspots” for a certain period of time [37]. For this paper, several synonyms were grouped, such as “decision making” and “choice”, and meaningless keywords were removed, such as “neuroscience” and “brain”. The frequency of keywords from 2000 to 2021 was calculated, as shown in Table 2. A total of 432 keywords were obtained from 2000 to 2021, but only 22 keywords appeared more than 15 times. Among them, “choice” ranked as the most frequent, indicating that existing research has focused on exploring the neuroscientific mechanisms induced by consumer decision-making. Concurrently, different cognitive and emotional functions correspond to different regions of the brain; the neural mechanisms induced by them have also received a high degree of attention, with corresponding keywords such as “emotion”, “attention”, “reward”, and “memory”. These were ranked 2nd, 3rd, 6th, and 15th, respectively. “fMRI”, which was ranked as the 13th most frequent, and “EEG”, which was ranked as the 19th most frequent, represent two types of important tools of neuroscientific research. The former provides feedback on the active areas of the brain by detecting changes in Blood Oxygen Level Dependent (BOLD) in the brain caused by neural activity. The latter measures change in EEG activity caused by neural activity through changes in electrical current frequency and voltage measured through the scalp [38]. There were two regions of the brain—the “prefrontal cortex” and “the orbitofrontal cortex”—that appeared in the word frequency statistics table, suggesting that most of the neural activity found in consumers in existing studies has involved these two regions. The former is associated with the emotion of closeness, while the latter is mainly associated with the emotion of rejection [39].

**Figure 3:**
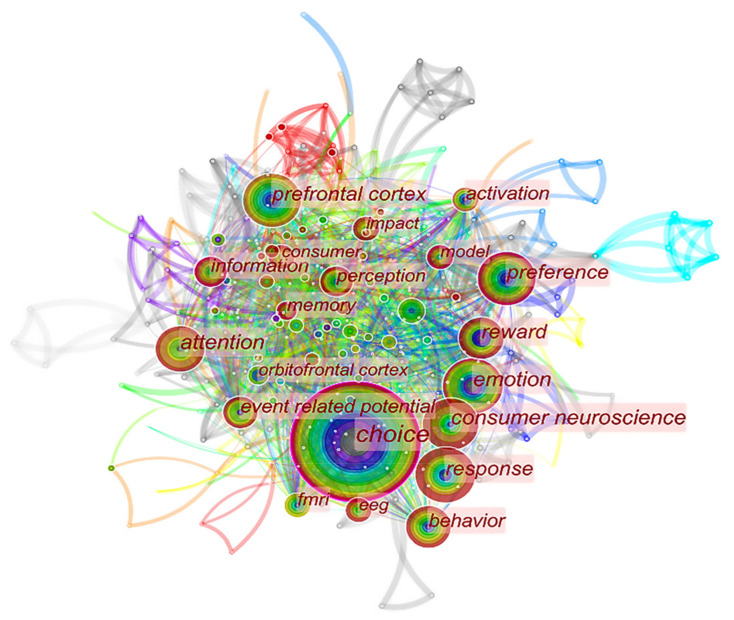
Keyword co-occurrence network.

Figure 3 shows the keyword co-occurrence network. The size of the circles represents the frequency of occurrence. The denser the linkage between nodes, the closer the relationship between keywords; the brighter the color of the linkage, the more research hotspots are derived. The largest circle in the network is that of “choice”, which is also the node with the highest centrality, connecting 55 nodes through it, including “emotion”, “attention”, “attention”, “reward”, “prefrontal cortex”, “memory”, and so on. This indicates that consumers’ decisions are related to unconscious processes such as emotion, attention, and memory [40]. Additionally, consumer decision-making is susceptible to the influence of preferences, with the greatest changes to feedback being those in the prefrontal cortex (PFC). It is worth noting that the nodes associated with “emotion” and “attention” are significantly different in terms of the choice of technical neuroscientific tools. The significant association of “fMRI” with the “emotion” node suggests that MRI is more frequently used in the research on consumer emotion. For example, the OFC is associated with positive emotions, while the lateral OFC and left dorsal anterior insula are associated with negative emotions [41]. The association of “EEG” with the “attention” node reflects the fact that EEG is more commonly used in studies on consumer attention. For example, consumers pay more attention to their preferred phone shape, showing significant increases in P200 and N200 magnitudes [42]. Based on consumers’ online shopping experience, Jones et al. found that positive evaluative information tends to elicit higher degrees of attention as well as greater P200 amplitudes [43]. Compared to traditional methods of cash payment when shopping, mobile payments tend to capture more consumer attention, corresponding to greater P200 amplitudes [28].

The keywords co-occurrence analysis based on CiteSpace mainly contains the following procedures: ① keywords frequency statistics through their extraction, separation, and classification; ② clustering similar research documents into a same cluster through their co-citation analysis [44].

**Figure 4:**
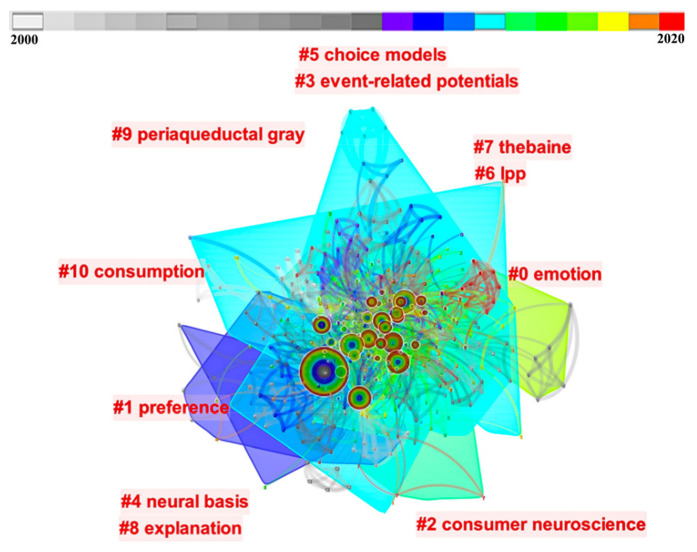
Clusters of related keywords.

A total of 12 clusters were identified through keyword clustering, which corresponds to silhouette values of 0.53 or more (clusters were considered reasonable if they were above 0.5). As shown in Figure 4, the largest cluster (#0) was that of “emotion”, emphasizing brain regions related to emotions. For example, Vecchiato et al. used EEG to investigate the emotional engagement of consumers watching TV commercials [45]. They observed that the left frontal and prefrontal lobe regions of the scalp were easily activated when participants experienced pleasure. Employing MRI technology, Hubert et al. found that product packaging with low levels of appeal easily activated brain regions associated with negative emotions [46]. Taking online payment as an example, if participants perceived online payments to be risky, brain regions associated with negative emotions were also activated, that is, the middle occipital gyrus [47].

The second major cluster (#1) was that of “preference”, which is based on neuroscientific techniques of exploring consumer preferences for products, brands, and advertisements, among others. Falk et al. reported that neural activity in the MPFC can effectively predict consumers’ purchasing decisions [48]. Using fMRI techniques, Van der Laan et al. showed that consumers’ preferred food packaging caused multiple regions such as the striatum to be strongly bilaterally activated [49]. Based on this, Golnar-Nik et al. found that EEG can assist consumers in effectively distinguishing between positive or negative preferences for advertisements, leading to increases in theta-band power in the left frontal region and right frontal region as preferences are enhanced and reduced, respectively [50]. The fourth cluster (#3) is that of “ERPs”, which focuses on the differences in brain-event-related potentials produced by different stimuli, involving P300, N200, P200, N400, LPP, and other electrical signals. Among them, P300 originally indicates the context updating that involves an attention-driven comparison process, now widely applied to observe the resource allocation regarding consumers’ attention and memorization [51]. Wang and Han found that consumer preferences lead to deviations between product utility and personal expectations, which in turn motivate consumers to generate more attentional resources and produce greater P300 amplitudes [52]. On this basis, consumer preferences also receive the interfering influences of external information, in that participants tend to think more about stranger-recommended information than friend-recommended information and call on more attentional resources, triggering greater P300 amplitudes as well as longer incubation periods [53]. N200 mainly signifies enhanced N200 amplitudes due to negative emotions. In branding studies, consumers produced increases in N200 amplitudes after being stimulated by photos with negative messages relative to photos with neutral messages [54]. In particular, “LPP” was presented separately within the cluster (#6), suggesting its particular importance in consumer neuroscience research. LPP is commonly used in emotional representation, with it being thought that changes in its amplitude relate to the regulation of emotions [55]. Bosshard et al. found that there was a significant correlation between brand name popularity and LPP amplitude, with preferred brands eliciting significantly more positive amplitudes than disliked brands [56]. This finding is interpreted as reflecting the positive emotions elicited by preferred brands. Goto et al. found that consumers were found to generate greater LPP amplitudes for preferred goods [57]. Cluster (#5) is the “choice model”, which stands for the consumer decision model. Neuroscience can reshape theoretical models of future consumption, as traditional decision models focus solely on the final choice of the consumer and do not fully reflect the unconscious evolution of the decision-making process [58]. Consumers’ unconscious processes tend to occur in the range of approximately 300 to 400 ms after the stimulus appears and are often difficult to detect through self-reporting methods such as questionnaires [59]. Neuroscience can fill such a gap, in that it can, through relevant variables (e.g., neural data related to cognitive processes such as emotion, attention, and memory), optimize the theoretical models of the influencing mechanisms underlying consumer decision-making [60].

### 3.5. Buzzwords

**Figure 5:**
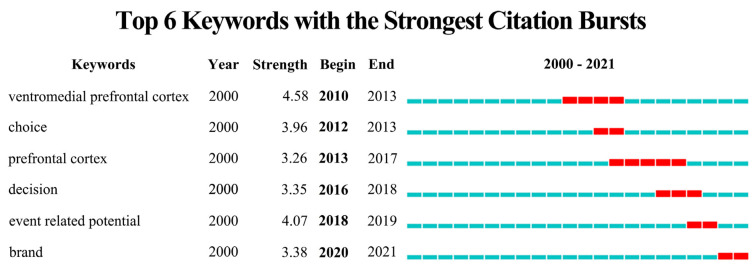
Top 6 keywords for burst detection.

Buzzwords are keywords that appear suddenly or increase in use significantly in a short period of time; they can direct future research [61]. Changes in buzzwords are generally based on keywords burst detection. A keyword ranking can be generated that is based on the frequency of occurrence of each keyword during the retrieved time period [37]. If the number of occurrences of a keyword increases significantly in a short period of time, it is indicated as a burst [20]. In Figure 5, the red rectangle indicates the time period with the highest degree of “burstiness”, and the “intensity” of this burst indicates that the keyword was mentioned significantly more frequently than other keywords in a given time period [62]. Six keywords in this study have significant burstiness. The first ranked term—“ventromedial prefrontal cortex”—and the third ranked—“prefrontal cortex”—became explosive buzzwords in 2012–2013 and 2013–2017, respectively. The above-mentioned articles also mention that the PFC is the most active region of the brain involved in consumer decision-making. The PFC is involved in regulating decision-making and payment behavior [63,64]. It is also closely related to consumers’ emotions and value assessments [65]. The keywords “choice” and “decision” exploded in frequency in 2012 and 2016, respectively. The aforementioned studies mention that, in consumer behavioral decision-making, consumers face the influences of unconscious processes such as emotions, attention, and memory [66]. Neuroscientific techniques can better fill such gaps and can even distinguish different mental processes of similar behaviors [67]. The explosion of “ERP” in 2018–2019 shows that as neuroscience techniques continue to advance, more studies are using single or multiple specific brain-evoked potentials (including P300, N200, P200, N400, and LPP) to observe changes in consumers’ cognitive responses to different marketing stimuli, such as advertising, price, packaging, etc. In addition, the explosion of the keyword “brand” between 2020 and 2021 illustrates the large number of studies focusing on marketing stimuli generated by this specific attribute of branding. Ma et al. found that, regarding brand familiarity, both brand familiarity and different product categories had significant effects on amplitudes of N270 as well as frontal electrode theta band power [68]. Meyerding and Mehlhose identified popular food brands using near infrared spectroscopy (fNIRS) [69]. They found that the more popular the brand, the higher the degree of PFC activation it resulted in.

## 4. Implications of Consumer Neuroscience towards Sustainable Consumption

The increase in individual environmental awareness may lead to a shift in consumption patterns towards sustainability. Especially since low-carbon development has become global common knowledge, consumer neuroscience has started to expand its research objects to include green brands, green labels, and green pricing [70,71]. Advertising, branding, packaging, and green labels are taken as external stimuli, and EEG indicators involved in consumer psychological feedback are taken as judgments of preference [72,73,74]. This is all in order to explore individual preferences for sustainable consumption. Looking at green branding with the use of EEG technology, it was found that theta activation was significantly higher in pro-environmental consumers than in those who were not [75]. Using fNIRS technology, Mehlhose and Risius revealed that products with organic labels attached caused increased activity in the PFC compared to products without these labels [76]. Herbes et al. found that the pricing of green electricity was predicted by neural activation metrics such as ERPs, and that WTP for green electricity was found to be approximately 15% higher than for non-green electricity [77]. Zubair et al. emphasized that ERP is an effective way to observe the effects of different emotional word stimuli in green advertisements, with both positive and negative emotional words tending to evoke changes in signals such as N1, P2, and LPP when consumers buy green products [78]. As for price information, non-pro-social consumers produced higher activation in the mPFC, bilateral anterior insula, cerebellum, anterior cingulate cortex (ACC), and inferior frontal gyrus compared to pro-social consumers [79]. Among these, mPFC regions were associated with reward and value, and the remaining regions were associated with decision-making [80]. This suggests that non-pro-social consumers may experience a greater sense of reward and sense of value for products marked with a price. In contrast, pro-social consumers are less likely to make purchasing decisions under price-only conditions and are also likely to focus on sustainability information on product labels, combining the attributes of both when making purchasing decisions [81]. The above findings help to motivate pro-environmental behavior based on consumer market segmentation. However, the number of articles published on green consumer neuroscience is still relatively small, less than 3% of the overall number of publications, reflecting the fact that there is still more room for subsequent development in this direction.

In addition, with the continuous development of data mining and analysis, relevant studies have begun to report on the use of machine learning and deep learning for the prediction of brain area changes relating to decisions of sustainable consumption and for the identification of brain area features [82]. Stanton et al. integrated and matched a large amount of experimental data with laboratory neuroscience data to predict brain regions associated with consumer decision-making [83]. Guixeres et al. analyzed the correlation between ERP, EEG power, and YouTube views by building an artificial neural network model [84]. Gholami Doborjeh et al. used a spiking neural network to analyze EEG data from consumers viewing familiar and unfamiliar trademarks and found that the model was able to identify brain activation patterns approximately 200 ms after stimulation [85].

In summary, this review provides the following insights into neuroscience application towards sustainable consumption: First, fNIRS technology, in addition to traditional EEG and fMRI technologies, has strong potential in discriminating whether consumer may prefer eco-labeled products, due to its advantages in allowing trials to immerse participants in a real consumption scenario as well as it also being less costly and more portable than fMRI [76]. Second, data analysis to neural signals through machine learning or deep learning techniques may be valuable to predict sales for eco-labeled products [86]. Moreover, the fusion of various physiological signal data, including EEG, eye movement electro-oculogram, hear rate, sphygmogram, etc., is expected to predict consumer choice towards sustainable consumption [84].

## 5. Discussion

In a comparison with the review literature published within the determined time period, the results of the keyword clustering analysis were found to be in strong agreement with the classification of the review literature [12,87]. In terms of research frameworks in consumer neuroscience, the focus is often on brain regions and ERPs associated with consumer cognition, as well as on different types of interventions and stimuli.

(1) Neural Responses in Specific Brain Regions

The high-frequency keywords identified in this paper using CiteSpace include “decision”, “reward”, “emotion”, “attention”, and “memory”, and are highly consistent with the classical review literature that focuses on their corresponding neural mechanisms. Keyword clustering revealed brain regions strongly associated with consumer cognition including the PFC and OFC, similar to those combed in the established review literature: ① brain regions associated with decision-making: PFC and OFC [88], ventromedial prefrontal cortex (VMPFC) [89], dorsolateral prefrontal cortex (DLPFC) [90], and ventrolateral prefrontal cortex (VLPFC) [91]; ② reward incentives: striatum and ventral tegmental area activation can reflect individual responses to rewarding stimuli [92,93]; ③ affiliation motivation and rejection motivation: A symmetrical activity over the frontal cortex has been implicated in the experience and expression of motivations [94]. The left frontal region is associated with approach motivation and, conversely, the right frontal region is associated with withdrawal motivation [95]; ④ emotion: activation in the amygdala and insula is associated with negative emotions produced by consumers [96,97]; ⑤ attention and memory: The PFC is responsible for directing and focusing attention, that is, the occipital lobe, while the hippocampus, located in the temporal lobe, is key to the acquisition of declarative memory [98,99]. Furthermore, consumers’ memories of familiar brands have been shown to activate the hippocampus [100,101]. The amygdala plays an important role in the consolidation of memory [102].

(2) The Main Event-Related Potentials of Interest in Consumer Neuroscience Research

① Decision-making: P300 is related to consumption decisions, and studies have found that both negative and positive comments elicit prolonged P300 [103,104]. ② Attention: N200 is associated with attention, and neural activity measured from the midfrontal electrode shows that the N200 component is associated with consumer attention and preferences of choice [57]. ③ Affiliation/rejection motivation: LPP can provide feedback on neutral, pleasant, or unpleasant stimuli [105]. For example, positive online shopping reviews can evoke greater LPP amplitudes in consumers [106], while feedback-related negativity (FRN) magnitude predicts consumer price sensitivity, with greater FRN magnitudes shown when prices are well below consumers’ expectations [107]. ④ Familiarity: N400 can detect the branding effect of a product, for example, putting Pepsi and footwear together can lead to a cognitive conflict and enhance N400 [26].

(3) Specific Marketing Incentives

In terms of bursting words, the brand is an important type of marketing stimuli and is of interest to consumer neuroscience. Additionally, related studies have used neuroscientific tools to observe the effects of interventions following a variety of marketing stimuli [108]. ① Brands: consumers’ memories of brands may result in brand preferences, for example, the mPFC is activated when subjects are confronted with familiar car brands [109]. ② Advertising: The degree of preference for advertising is reflected by observing brain activity while viewing advertisements. More popular advertisements tend to elicit higher degrees of neural activation [110]. ③ Price: Prices exceeding consumer expectations may lead to brain activation [111]. This is particularly the case in certain products with high added value; the premium price may affect consumer-perceived potency, for example, wine with a higher price tag may elicit higher activation in the mOFC [112]. ④ Appearance: It includes packaging, color, labeling, and all other elements that can be visually perceived by the consumer [113]. For example, the styling of a luxury sports car may evoke more significant activation in the striatum and orbitofrontal cortex in the brain than a car with only basic styling [114]. ⑤ Social influences: This includes the potential impact on consumers of other people’s comments, social morals, etc. [115]. For example, positive online reviews cause consumers to engage in cognitive processing, thus producing greater LPP amplitudes [116].

(4) Consumer Neuroscience Technology

**Figure 6:**
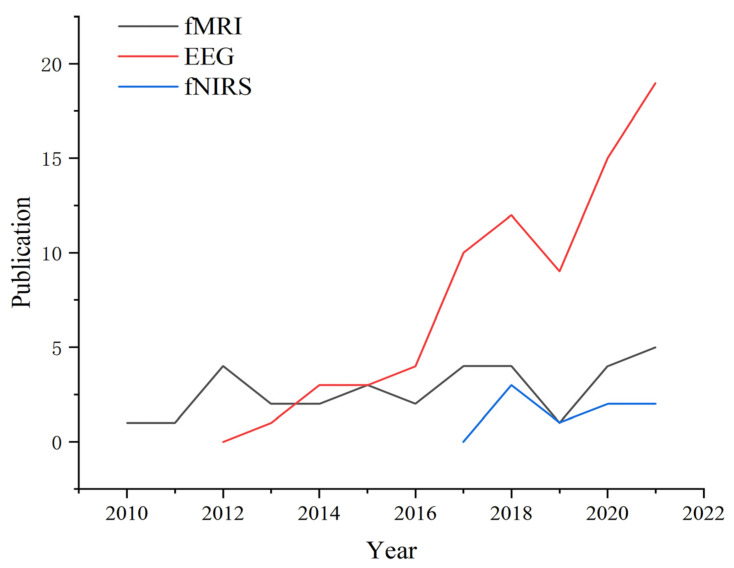
Changes in neuroimaging research tools, 2010–2022.

In terms of research tools for neuroscience, statistics during the search period found that 63.8% of studies chose to use EEG, 29.8% used fMRI, and 7.4% used fNIRS. Of particular note is the fact that the number of papers using EEG techniques for consumer research since 2015 exceeded those using fMRI techniques and this shows a trend of continuous increase, as shown in Figure 6. A possible reason is that EEG is a relatively less invasive and less costly method of scalp-recorded brainwave measurement. It provides high temporal resolution (250–400 ms) and helps researchers understand the cognitive and emotional processes involved in consumer decision-making [117]. However, its low spatial resolution (~1 cm^3^) does not allow for the identification of the specific location of brain activity, which may lead to the production of the same potentials at different location sources [118]. fMRI can detect metabolic changes in blood oxygen flow in the brain originating from neural activation, which is used to reveal areas of brain activation associated with consumer emotion and memory [119]. In addition, research papers based on fNIRS technology have started to emerge since 2018, with 1–3 relevant articles published each year until 2021. fNIRS has a spatial resolution and penetration depth of up to 2 cm and is suitable for monitoring brain regions located in the PFC [120]. It is also less costly and more portable than fMRI [121].

Neuroscience can play an effective role in better demonstrating those unexplained variables, including attention, attitude, preference, willingness to pay, etc. [17]. We have noted that there are reports on the advertising performance test by neuroscience measures compared with field experiments. For example, fMRI was used to test the validity of different television campaigns on smoking cessation compared to that predicted by field investigations, indicating that there is a distinction between neural responses with behavioral responses [48]. Bellman et al. took the inter beat interval (ibi), skin conductance, and facial expression as the observational variables to discriminate effective or ineffective television advertisements via in-market sales response [122]. In such contexts, it is suggested that neuroscience is powerful for revealing the heterogeneity between consciousness and behavior [123]. For instance, it is expected to provide a better understanding of why people disregard environmental impacts when they are intended to buy one-off plastic bags for food packaging, although they have been aware of such environmental and health issue [124]. The insights of this new area have the potential to shed new light on studies on consumers’ purchasing behavior regarding environmentally friendly products, and how to influence consumers effectively by using different forms of eco-labeling presentation.

## 6. Conclusions

This study reviewed the progress of consumer neuroscience research during the period 2000–2021 based on bibliometric analysis. Through statistical analysis of the number of published articles, countries of publication, institutions of publication, and keywords, we analyzed research trends in the field, identified research hotspots, and provided insights into the integration of neuroscience into research on sustainable consumption. Our results show that a total of 364 publications were published during the identified search period, showing a significant increase, reflecting the fact that the application of neuroscience tools in the field of consumption is expanding. Consumer neuroscience research is dominated by research institutions in the United States and China, with the California Institute of Technology in the United States and Zhejiang University in China having the highest number of publications. Respectively, these focus on the use of fMRI and EEG techniques to explore the neural mechanisms of consumer decision-making. Through the identification of keywords and buzzwords, it was found that the research is largely focused on ERP studies based on different marketing stimuli; consumer decision-making and emotion-specific brain regions based on fMRI techniques; and decision model optimization studies based on electrophysiological signals.

Through bibliometric analysis, this paper found that existing studies have focused on neural features related to consumer cognitive processes, including neural responses and ERPs in specific brain regions. This is followed by a focus on consumers’ neural responses to specific marketing stimuli, mainly by examining marketing stimuli such as advertising, branding, price, and packaging. In addition, the majority of consumer neuroscience studies have chosen to use EEG tools, accounting for 63.8% of the total number of studies published. One-third of the studies used fMRI. Furthermore, studies using fNIRS techniques have begun to emerge in recent years.

The research applications of neuroscience in sustainable consumption should continue to expand. This can be accomplished by, firstly, analyzing indicators such as EEG signals or brain region activation to make predictions on consumers’ decision-making and their potential cognitive processes. Secondly, based on data mining and analysis techniques, machine learning and other means can be used to optimize consumers’ preferences—or decision models—for green products/services. In addition, eye movement, heart rate, pulse, and other psychological indicators could also be integrated in order to perform a comprehensive judgment of consumer decision-making. Concurrently, questionnaires or in-depth interviews could be used as comparisons to reflect the difference between individual and group decision-making. There is room for improvement with the review. It is expected that bibliometric analysis may be merged with systematic review via PRIZMA statement that provides useful criteria for the article selection, to ensure review quality while not missing any classic literature.

## Figures and Tables

**Table 1:** Institutions with the largest number of publications.

Institution	Country	Number of Publications
Zhejiang University	China	14
California Institute of Technology	United States	10
Erasmus University	Netherlands	9
Zhejiang University of Technology	China	9
Ningbo University	China	8
Duke University	United States	8
University of Granada	United States	8
University of Michigan	United States	8
Guangdong University of Technology	China	7
Columbia University	United States	7

**Table 2:** Descriptive statistics for keywords.

Keyword	Centrality	Number of Publications	Percentage (%)
choice	0.29	90	21.18
emotion	0.16	41	9.65
attention	0.08	41	9.65
consumer neuroscience	0.07	41	9.65
response	0.06	38	8.94
reward	0.09	38	8.94
preference	0.11	36	8.47
prefrontal cortex	0.09	35	8.24
event-related potential	0.04	33	7.76
behavior	0.07	29	6.82
information	0.12	28	6.59
activation	0.14	28	6.59
fMRI	0.07	27	6.35
perception	0.04	27	6.35
memory	0.12	23	5.41
model	0.14	21	4.94
consumer	0.06	21	4.94
impact	0.02	21	4.94
EEG	0.02	20	4.71
orbitofrontal cortex	0.05	20	4.71
choice	0.06	17	4.00
emotion	0.02	16	3.76

## Data Availability

No new data were created or analyzed in this review. Data sharing does not apply to this article.
